# Effects on Transcriptional Regulation and Lipid Droplet Characteristics in the Liver of Female Juvenile Pigs after Early Postnatal Feed Restriction and Refeeding Are Dependent on Birth Weight

**DOI:** 10.1371/journal.pone.0076705

**Published:** 2013-11-19

**Authors:** Constance Nebendahl, Ricarda Krüger, Solvig Görs, Elke Albrecht, Karen Martens, Steffen Hennig, Niels Storm, Wolfgang Höppner, Ralf Pfuhl, Barbara U. Metzler-Zebeli, Harald M. Hammon, Cornelia C. Metges

**Affiliations:** 1 Institute of Nutritional Physiology ‘Oskar Kellner’, Leibniz Institute for Farm Animal Biology (FBN), Dummerstorf, Germany; 2 Institute of Muscle Biology and Growth, Leibniz Institute for Farm Animal Biology (FBN), Dummerstorf, Germany; 3 ImaGenes GmbH, 13125 Berlin, Germany; 4 Bioglobe GmbH, 22529 Hamburg, Germany; University of California Davis, United States of America

## Abstract

Epidemiological and experimental data indicate that caloric restriction in early postnatal life may improve liver lipid metabolism in low birth weight individuals.

The present study investigated transcriptional and metabolic responses to low (U) and normal (N) birth weight (d 75, T1) and postnatal feed restriction (R, 60% of controls, d 98, T2) followed by subsequent refeeding until d 131 of age (T3). Liver tissue studies were performed with a total of 42 female pigs which were born by multiparous German landrace sows. Overall, 194 genes were differentially expressed in the liver of U vs. N (T1) animals with roles in lipid metabolism. The total mean area and number of lipid droplets (LD) was about 4.6- and 3.7 times higher in U compared to N. In U, the mean LD size (µm^2^) was 24.9% higher. 3-week feed restriction reduced total mean area of LDs by 58.3 and 72.7% in U and N, respectively. A functional role of the affected genes in amino acid metabolism was additionally indicated. This was reflected by a 17.0% higher arginine concentration in the liver of UR animals (vs. NR). To evaluate persistency of effects, analyses were also done after refeeding period at T3. Overall, 4 and 22 genes show persistent regulation in U and N animals after 5 weeks of refeeding, respectively. These genes are involved in e.g. processes of lipid and protein metabolism and glucose homeostasis. Moreover, the recovery of total mean LD area in U and N animals back to the previous T1 level was observed. However, when compared to controls, the mean LD size was still reduced by 23.3% in UR, whereas it was increased in NR (+24.7%).

The present results suggest that short-term postnatal feed restriction period programmed juvenile U animals for an increased rate of hepatic lipolysis in later life.

## Introduction

Intrauterine growth retardation (IUGR) is a frequent cause of perinatal morbidity which prevents the fetus from meeting its optimal growth potential [1]. IUGR is associated with impaired growth during childhood [2] as well as metabolic diseases in adulthood [3], [4]. Overall, epidemiological studies show a convincing link between low body weight at birth and increased propensity for adult diseases [5]–[7]. From human studies, the most common definition of IUGR is a fetal weight below the 10th percentile for gestational age (birth weight <2.5 kg). Individuals born small for gestational age (SGA) show lower insulin sensitivity and higher abdominal fat mass, and more often disturbed lipoprotein levels and increased liver lipid content [8], [9]. One study in male IUGR mice showed elevated triglyceride levels, which were attributed to increased hepatic fatty acid synthesis and decreased beta-oxidation [10].

It is well known that caloric restriction may prevent late-onset metabolic diseases such as hyperlipidemia and diabetes mellitus [11], [12]. These effects were attributed to improvements of liver lipid metabolism and stress responses [13]–[15]. Based on these data and the fact that inadequate intrauterine conditions may alter hepatic energy and lipid storage state [9], [16], liver tissue seems to be of interest for studying effects linked to fetal growth reduction. Because metabolic imprinting occurs during critical periods such as prenatal life [17], long-term alterations of gene expression may lead to persistent effects of IUGR on liver lipid homeostasis. Overall, there is growing evidence that IUGR alters the epigenetic state of the fetal genome and imprint gene expression [18], [19]. Two mechanisms that underlie these epigenetic effects are DNA methylation and histone modification [20], [21].

Based on these previous observations, we investigated IUGR and normal birth weight female porcine offspring before and after postnatal feed restriction and a subsequent refeeding period in regard to hepatic molecular and physiological changes. The liver is the central metabolic organ, integrating nutrient intake and supply to the peripheral tissues. The pig is increasingly used as a biomedical animal model due to its similarity to human physiology [22]. Porcine offspring shows up to 20% of naturally occurring IUGR [23] leading to fetal metabolic aberrations, reduced postnatal growth and increased body fatness during puberty and young adulthood [24], [25]. It has been also shown that early postnatal catch-up growth in low birth weight piglets was associated with insulin resistance in adult pigs [26]. In addition, in small porcine fetuses a delayed adipocyte differentiation as compared to normal weight fetuses and lower body fatness at birth has been observed [27].

In our study, the following questions were addressed at the transcriptional, epigenomic and metabolic level: 1) Are there differences in the hepatic transcriptional profile between IUGR and normal birth weight pigs? 2) Are these effects reflected on the metabolic level? 3) Could potential birth weight-dependent effects be modified through feed restriction intervention? 4) For how long do these effects persist after refeeding and, moreover, are there alterations in variables of lipid homeostasis? To our knowledge, there are no reports on effects of feed restriction and subsequent refeeding periods on liver lipid metabolism in IUGR vs. normal birth weight adolescent mammals. However, there is evidence from human studies that impaired fetal growth can induce long-term effects on ontogenetic development and disease state [28], [29].

## Materials and Methods

### Animal selection and treatment

Procedures performed in this study were in accordance with the German animal protection law and approved by the Landesamt für Landwirtschaft, Lebensmittelsicherheit und Fischerei, Mecklenburg-Vorpommern, Germany (LALLF M-V/TSD/7221.3-1.1-049/09). Liver tissue studies were performed with a total of 42 female pigs which were born by multiparous German landrace sows with a mean litter size of 15 piglets. Littermates were selected for low (0.8–1.1 kg, U) or normal (1.4–1.6 kg, N) birth weight. From birth until weaning at d 28, U and N piglets were suckled by their dams with no creep feed provided. After weaning, piglets were housed individually and had free access to water and feed. Feed intake was determined daily and body weights were monitored at weekly intervals. Different diets were fed during the growing period to meet or exceed the nutrient intake recommendations according to the respective growing stage (GfE, Society of Nutritional Physiology, 2006). After weaning, piglets were fed a diet containing a mixture of 50% (w/w) commercial pig feed (22% crude protein; BabyCrisb EW, Bergophor-Futtermittelfabrik, Kulmbach, Germany), 30% rolled oats (12.5% crude protein; Holstenmühle, H. & J. Brüggen KG, Lübeck, Germany), and 20% sucrose (Nordzucker, Braunschweig, Germany) with a total energy content of 16.9 MJ ME/kg (16.2% crude protein, 98% dry matter). From d 78 until d 98 half of the U and N pigs were subjected to feed restriction (60% of *ad libitum* consumption) of a conventional pig diet (Vormast CAFO TOP, Trede & von Pein Landhandel und Mischfutterwerk, Dammfleth, Germany; 14.7 MJ ME/kg; 19.7% crude protein). Subsequently, both restricted (UR, NR) and control groups (UK, NK) were subjected to *ad libitum* feeding until d 131 (75% Vormast CAFO TOP, 15% rolled oats, and 10% sucrose; 15.2 MJ ME/kg; 16.5% crude protein). Overall, four pig groups (NK, NR, UK, UR) were included in our study to analyze effects related to birth weight (U vs. N) and/or feed restriction (R vs. K). Liver tissue was taken from animals after overnight fasting (18 h) at ages d 75 (before feed restriction, T1), d 98 (after 3 week feed restriction, T2), d 104 (after 5 d of refeeding, T2.1) and d 131 (after 5 weeks of refeeding, T3). Animals were killed by electrical stunning and exsanguination and livers were removed within 10 min after death. Liver studies were performed on three animals per group at each time point.

### Whole genome expression profiling and bioinformatics analysis

Whole genome expression studies were performed on liver tissues of three randomly chosen animals per group at ages d 75 (T1), d 98 (T2) and d 131 (T3). Total RNA was isolated from the liver with the RNeasy Mini Kit (Qiagen, Hilden, Germany). After quantification, RNA aliquots of each animal were hybridized to porcine-specific Agilent 8×60 K multiplex arrays (Agilent, Santa Clara, USA). After quantile normalization of microarray data, statistical analysis for pairwise comparisons was performed with a paired t-test with unequal variance. Normalized intensity levels for each of the 19,864 transcripts were used to calculate mean expression values and fold changes for all compared groups. Pairwise comparison analysis resulted in increased, decreased, not changed or not detected gene expression levels. A gene list was selected applying the following criteria: thresholds for gene expression levels ≥1.3 with p-value≤0.05. For the classification of regulated genes according to their functional roles, and to identify common pathways between these genes, further bioinformatics analyses were conducted by using DAVID bioinformatics and Ingenuity pathway analysis (IPA). The complete microarray data sets and information about study design and methodology were submitted to Minimal Information about Microarray Experiments (MIAME) with the GEO accession number GSE43826 (http://www.ncbi.nlm.nih.gov/geo/query/acc.cgi?token=prqfbucseomeyvo&acc=GSE43826).

### Oil red o staining and lipid droplet analysis

For histologic analysis, frozen liver tissue of UK, UR, NK, and NR pigs at three time points (T1, T2, T3) were cut into 10 µm sections and fixed with formol-calcium for 5 min. Thereafter, sections were rinsed in distilled water and neutral lipids were stained with Oil Red O for 15 min. Intracellular lipid droplets (LD) were detected as red spheres in liver tissue sections using computerized image analysis. The image analysis system was equipped with a Jenaval microscope (Carl Zeiss, Jena, Germany), an Altra20 color camera (OSIS, Munster, Germany), and CELL∧D image analysis software (OSIS, Munster, Germany). The sequence of analysis steps was organized in a newly developed macro program. The sequence was as follows: At first, the color image was taken, the green channel was extracted and preprocessed to enhance the contrast and to improve the detectability of LD. A threshold was interactively determined for discrimination between background and objects (LD) to be measured. An interactive step was included to delete false detected objects like artifacts. The total number and area of LD in the selected region, area percentage occupied by LD, individual and mean LD size and distribution of LD as distance between neighbors were determined. The results presented are means ± SEM of three animals per experimental group with 16 observations per animal (yielding a total of 48 observations per experimental group).

### Promoter DNA methylation analysis

Methylation studies were performed on liver tissue DNA of UKT1, NKT1, UKT2, URT2, NKT2, NRT2, UKT3, URT3, NKT3, NRT3 pigs (n = 3 per group). Genes with changed expression profiles and publicly available sequence annotations were selected as candidate genes (PTPRS, PDX1, PPP1R3E, SORT1, WNT5B, SFRP4, SAG, PDE9A, FGFR4, FABP5). Most promising target regions of highest CpG density (CpG islands) within the putative promoter region (up to 2000 base pairs upstream of the transcription start) were determined with EBI's open access tool Cpgplot (http://www.ebi.ac.uk/Tools/seqstats/emboss_cpgplot/). Except for FGFR4, the first transcribed exons contained increased CpG-density as well. Therefore, these regions were included in the analysis targets.

Quantitative methylation analysis was performed with the MassARRAY® system (Sequenom, Hamburg, Germany).

Assay design was aided by platform specific software EpiDesigner, accessible through Sequenom's customer page www.mysequenom.com. The software divides the region of interest into suitable amplicons and delivers information on primer sequences and positions, amplicon size, and CpG-coverage. Where possible, assay design/analysis was performed for forward as well as reverse strand of the genomic DNA sequence to ensure best CpG-coverage and system-inherent confirmation with a second independent reaction. The design outcome is summarized in table 1.

**Table 1:** PCR primers used for the analysis of the methylation status of selected gene promoters.

Selected amplicons	Direc-tion*	Chro-mo-some	Start relative to gene	End relative to gene	Amplicon length	Amplicon CpGs	Left primer (+Tag)#	Right primer (+Tag)#
PTPRS_amp03	F	2	−2509	−2162	348	9	aggaagagagTTGGTTATTTTTGTTTTTAAAGGGTT	cagtaatacgactcactatagggagaaggctAAAAAAAACATCCACACAAAATCAC
PTPRS_amp09	F	2	−2186	−1700	487	8	aggaagagagGTGATTTTGTGTGGATGTTTTTTTT	cagtaatacgactcactatagggagaaggctTAATTCTAACTCCCAAAATACCCAA
PTPRS_amp12	F	2	−1873	−1545	329	10	aggaagagagGGTTTGATTGTGTGTTTTTTGAGAT	cagtaatacgactcactatagggagaaggctAATTTCCACTTTAACCAAAACCAA
PTPRS_amp16	F	2	−1570	−1099	472	20	aggaagagagGTTTGGTTTTGGTTAAAGTGGAAAT	cagtaatacgactcactatagggagaaggctATTTAACCACCAAATAAATCTTCCC
PTPRS_amp17	F	2	−1226	−890	337	11	aggaagagagTGGGGTTATGTGGTTGTATTTTTTA	cagtaatacgactcactatagggagaaggctAACCTACACCACAACTCAAATCAAT
PTPRS_amp25	F	2	−322	122	444	15	aggaagagagGAGGGTTAGGTTTTTATATAATTTTGGA	cagtaatacgactcactatagggagaaggctAACACACACAAAACCAAATACTCAC
PTPRS_amp30	R	2	−2486	−2081	406	11	aggaagagagAGGGTTATAGTGGGGTAGATAATGT	cagtaatacgactcactatagggagaaggctACCCAAAATAAAAACACAAAACAAA
PTPRS_amp37	R	2	−1561	−1326	236	7	aggaagagagGTTGTTATAATGGGAATTTTTGAAT	cagtaatacgactcactatagggagaaggctAAAACCATATAACTACACCTCCCAC
PTPRS_amp41	R	2	−1033	−730	304	9	aggaagagagGGGATTAGGGTTTTTATAAAGGGAT	cagtaatacgactcactatagggagaaggctTCAAAACTAAAATCTAAACTTCATCACAA
PPP1R3E_amp01	F	7	−87	135	222	12	aggaagagagGGGGAGGTAGAGTGGTTTTATTTT	cagtaatacgactcactatagggagaaggctCACTAACCTTCAACTTCCAAAATCT
PPP1R3E_amp07	F	7	−12	135	140	11	aggaagagagATGGGGAGTTATTTGTTAGAGGGTA	cagtaatacgactcactatagggagaaggctCTTCAACTTCCAAAATCTTAAACCA
PPP1R3E_amp12	F	7	188	833	646	72	aggaagagagTTGGAGGTGATAGTAAGGAGAGTAAGAAAT	cagtaatacgactcactatagggagaaggctCAAAAACCAACCCAAAATTAAAAAT
SORT1_amp04	F	4	−702	−197	506	20	aggaagagagTTTTTGTAGAAGAGGAAGATGAGTGA	cagtaatacgactcactatagggagaaggctCCTCCTACTTAAAAAATCCTAAATTC
WNT5B_amp24	R	5	−1735	−1369	367	9	aggaagagagTTTGGGATTAGAGATGGAGTAGAGA	cagtaatacgactcactatagggagaaggctACTATCCTAAAAAACAAAAACCCCA
WNT5B_amp28	R	5	−1180	−821	360	11	aggaagagagATTTTGGAGTAAGTTTTTGGAAAGG	cagtaatacgactcactatagggagaaggctCACACACCTACCTATTAATACCCCC
WNT5B_amp31	R	5	−845	−462	384	10	aggaagagagTGGAGTTGAAAGTAATTGGTAAGTTG	cagtaatacgactcactatagggagaaggctCCTTTCCAAAAACTTACTCCAAAAT
WNT5B_amp38	R	5	−487	−166	322	8	aggaagagagGGAGAGTTTGTTTTTTTAGGAAAGG	cagtaatacgactcactatagggagaaggctCAACTTACCAATTACTTTCAACTCCA
PDE9A_amp05	F	13	−1855	−1392	464	11	aggaagagagGGAGGGTTTAGGGAGATATTTGATA	cagtaatacgactcactatagggagaaggctCAAATAAAAACAAAAAACCCCTCTT
PDE9A_amp14	F	13	−1342	−890	453	20	aggaagagagGGAGGTAGAGGGGGTTGTTAGTTAG	cagtaatacgactcactatagggagaaggctTTAACCCACAAAAAAAACTATCCC
PDE9A_amp15	F	13	−1018	−680	339	22	aggaagagagTTTTGTTGTGTTGATATTTGTTTGG	cagtaatacgactcactatagggagaaggctAAAAACTAACAACTTTTATATAACCCC
PDE9A_amp17	F	13	−608	−202	407	12	aggaagagagTTTGTTTTTTGAGAGTTTGGTAAAG	cagtaatacgactcactatagggagaaggctTCAAACCAAAAACCATAAAATTACAA
PDE9A_amp24	R	13	−2258	−1831	428	35	aggaagagagTGTTAAGTGTTTTTTTGGGTTTTTT	cagtaatacgactcactatagggagaaggctCCTCAACATAAACTTATCCATCCTC
PDE9A_amp25	R	13	−1907	−1548	360	11	aggaagagagTGTATTGGTTATAGAGTTGGGAGGA	cagtaatacgactcactatagggagaaggctATCACAAACCAAACCAAACTCC
PDE9A_amp34	R	13	−1572	−1129	444	13	aggaagagagGTGGTTGGTTAGGGTTTTTTTTAAG	cagtaatacgactcactatagggagaaggctTCCTCCCAACTCTATAACCAATACA
PDE9A_amp41	R	13	−230	63	293	14	aggaagagagTTATTGAAGGTAAAAGTAGGGTTGG	cagtaatacgactcactatagggagaaggctAACCTACAACCCCATAATTCCTAAC
FGFR4_amp04	F	2	−2675	−2178	498	10	aggaagagagTGGTTTTGAGAGGTGTAGTTATTTG	cagtaatacgactcactatagggagaaggctAACAAAACCCAATCCACACTATTTA
FGFR4_amp26	R	2	−2989	−2740	350	22	aggaagagagGGGATTTTGGATAAATGATTGTATTT	cagtaatacgactcactatagggagaaggctCCTCCCAACACTAAAAACCTAAAC
FGFR4_amp38	R	2	−2013	−1677	337	5	aggaagagagTATTATTTGGGGTTGGGTAATAGGT	cagtaatacgactcactatagggagaaggctCCCAAACTTATCATTTTTAAAACCC
FGFR4_amp44	R	2	−1392	−893	500	5	aggaagagagGGGTTAAATTTAGGGTTAGGGGTTA	cagtaatacgactcactatagggagaaggctATCCTCAAAATAATCCTAAAAAAAA
FGFR4_amp46	R	2	−903	−494	410	6	aggaagagagTTTGGATGTTATTTGATTTGATTTT	cagtaatacgactcactatagggagaaggctAAATTTAACCCCTAACCCAAAAACT
FABP5_amp09	F	4	217	701	485	28	aggaagagagGTGTTGGGTTTAGGGGTTAGGTAG	cagtaatacgactcactatagggagaaggctAAAAACCTAATCCAAACTCTCTAAAAAA
FABP5_amp14	F	4	670	1068	399	23	aggaagagagGAAATTTTTTAGAGAGTTTGGATTAGG	cagtaatacgactcactatagggagaaggctAATTAAAAAACAACCCCAAAAAAAC
FABP5_amp19	F	4	1123	1495	373	4	aggaagagagTTAAGGATGAGTGATTTTTAGGTAGGA	cagtaatacgactcactatagggagaaggctCAAAAAACCAACCTTAACCAACTAA
FABP5_amp28	R	4	−987	−562	426	5	aggaagagagTTTAGTTTAATTGGGGTTTTTGGAT	cagtaatacgactcactatagggagaaggctTTACCTCCACCAAAATAACTACCCT
FABP5_amp29	R	4	−656	−336	321	8	aggaagagagATGTTTTTTTTGGGTTAGAGGATTT	cagtaatacgactcactatagggagaaggctCTTCCTAAAACTTTTCCAACTACCC
FABP5_amp30	R	4	−361	91	452	48	aggaagagagATTAGTTGTTGGATGGAGGTTATGG	cagtaatacgactcactatagggagaaggctTAAATCCTCTAACCCAAAAAAAACA
FABP5_amp33	R	4	189	618	430	22	aggaagagagTTAGGAATAAAAGATGTTGTAGGGTG	cagtaatacgactcactatagggagaaggctAACTATATCCCTAAAACACCCCCTC
FABP5_amp38	R	4	1044	1425	382	5	aggaagagagGGATTTAGGTTTTTGTAGTGATTTTTG	cagtaatacgactcactatagggagaaggctACCCTCCTAAAACTATTTTCTAACTAA

All sequences are given in 5′→3′ direction of the analytical PCR product.

* F, forward; R, reverse,

# Tags are written in lower case; left primer contains a 10mer tag sequence for annealing adjustment, right primer contains a 31mer tag consisting of T7-RNA-polymerase promoter sequence and an 10mer spacer sequence.

The MassCLEAVE™ biochemistry was applied after bisulfite treatment of DNA samples and MALDI-TOF mass spectrometry for analyte detection according to the standard protocols recommended by the supplier. Genomic DNA was extracted from pig liver with the DNeasy Kit (Qiagen, Hilden, Germany). One µg DNA was treated with sodium bisulfate bisulfite (DNA EZ Bisulfite Treatment Kit, Sequenom, Hamburg, Germany) according to supplier's manual to covert Cytosin to Uracil at non-methylated CpG-sites whereas each 5-Methyl-Cytosin persists as Cytosin. Regions of interest were amplified by PCR from bisulfite treated DNA samples using methylation independent primers (Metabion International, Martinsried, Germany; Table 1). PCR products were then subject to simultaneous in vitro transcription and RNase A cleavage applying the T-reverse reaction following Sequenom's recommended standard protocol. The generated fragments were displayed based on their molecular weight in the mass spectrum, which was acquired after sample conditioning with a MassARRAY® Analyzer Compact. The resulting methylation calls were analyzed with EpiTyper Software (Sequenom) to generate quantitative results for each CpG site.

Statistical analysis followed a boot-strap method where first, the methylation ratios differences between experimental and control group are determined. Then, samples were randomly reassigned to experimental and control group and the methylation difference calculated again. Finally, the data display statistical significance if the determined methylation differences persist in the true compared to the random groups.

### Liver amino acid analysis

For amino acid analysis, liver tissue from 8 experimental groups (UKT2, URT2, NKT2, NRT2, UKT3, URT3, NKT3, NRT3) was included (n = 3 per group). In detail, 20 mg of each liver sample was homogenized in 60 µl lysis buffer containing 50 mM Tris (pH 7.8), 1 mM EDTA (GE Healthcare, Munich, Germany), 10 mM NaF (Fisher Scientific, Schwerte, Germany), 1% (v/v) Igepal CA-630 (Sigma-Aldrich, Taufkirchen, Germany), 0.1% (v/v) Triton X-100 (GE Healthcare, Munich, Germany), 0.5% (v/v) deoxycholic acid (DOC; Sigma-Aldrich), 0.1% (w/v) sodium dodecyl sulfate (SDS; USB Corporation, Cleveland, OH, USA) and Roche Phospho-Stop tablets (one tablet/10 ml buffer; Roche Diagnostics, Mannheim, Germany). Protein concentrations in solubilized homogenates were determined by Bradford assay (Sigma-Aldrich, Germany). Liver homogenates from three animals per group were diluted with water (1∶20) and free amino acids were analyzed by HPLC equipped with a fluorescence detector (Series 1200, Agilent Technologies, Germany). The HPLC analysis method was adapted from the technique described by Krömer et al. [30]. Briefly, amino acids were separated after automated pre-column derivatization with ortho-phthalaldehyde/3-mercaptopropionic acid and 9-fluorenylmethoxycarbonyl chloride after reaction with 3-mercaptopropionic acid as reducing agent and iodoacetic acid to block sulfhydryl groups. Analyses were carried out at a flow rate of 0.8 ml/min within 45 min on a 250×4 mm Hyperclone ODS (C18) 120 Å column protected by a 4×3 mm C18 pre-column (Phenomenex, Aschaffenburg, Germany) using a gradient with 40 mM phosphate buffer (pH 7.8) and acetonitrile/methanol/water (v∶v∶v: 45∶45∶10) ranging from 6–100%.

### Statistics

Statistical evaluation was done with SigmaPlot 11.0 software (Systat Software GmbH, Erkrath, Germany). For four-group comparisons (UK, NK, UR, NR), Two-Way Analysis of Variance (ANOVA) with the factors birth weight and feed restriction was used. When data passed the normality test (p≤0.05), Bonferroni-test was used to detect group differences. Not-normally distributed data were log-transformed to obtain normality and to allow ANOVA analysis. For two-group comparisons of gene expression data, paired Student's t-test was used. Results are depicted as means ± SEM, and were considered significantly different when p-values were p≤0.05. Trends were discussed when 0.05≤p≤0.1.

## Results

### Effects of feed restriction and three weeks refeeding on body weight development in low (U) and normal (N) birth weight pigs

**Figure 1:**
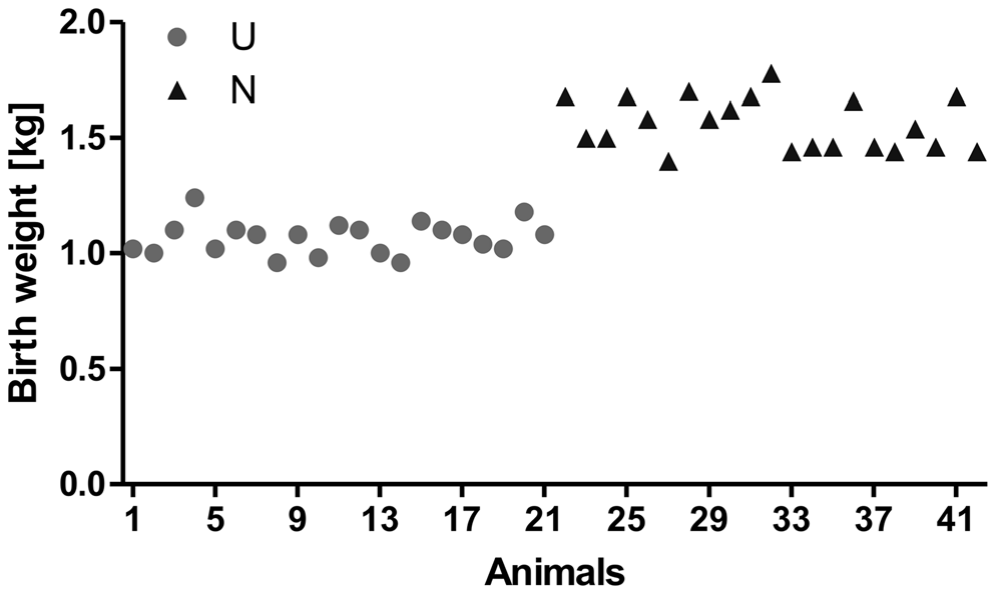
Birth weights of N and U piglets used for liver tissue studies. With regard to birth weight, piglets were classified into low (U) and normal (N) birth weight groups with 21 animals each. There was a difference (p≤0.001) in body weight between U and N animals with 1.07±0.02 and 1.56±0.02 kg, respectively.

**Figure 2:**
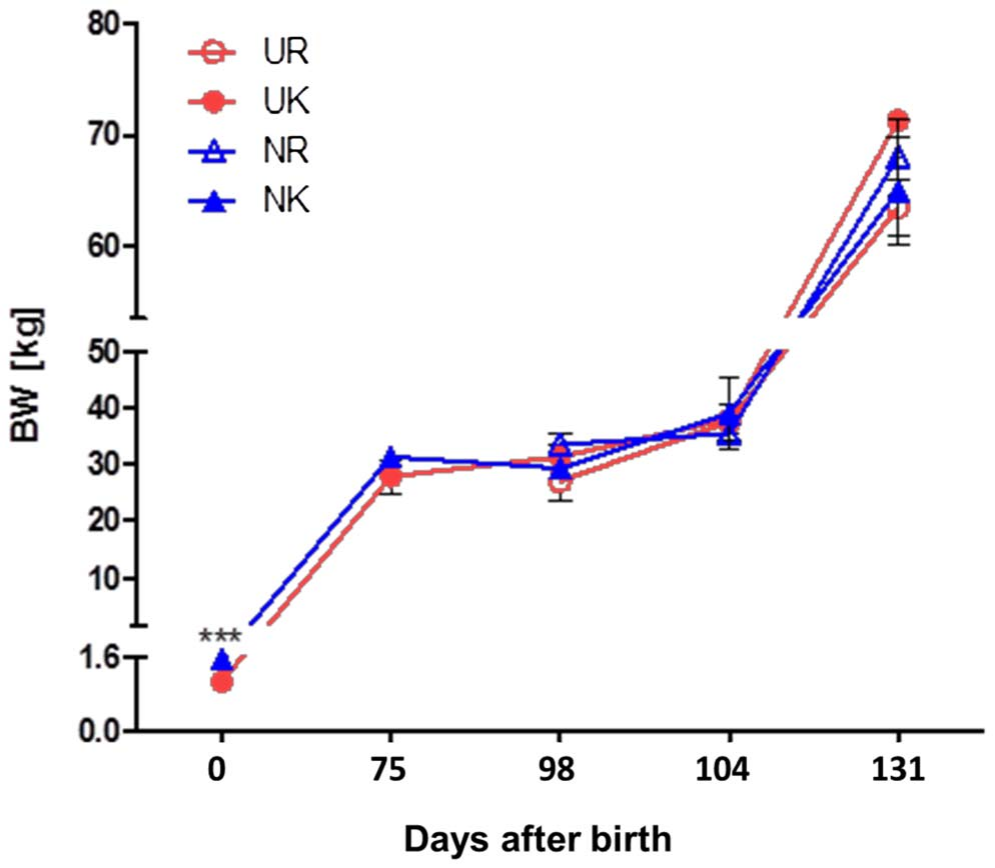
Body weight development of U and N pigs after feed restriction (T2) and refeeding (T3). Pigs with low (0.8–1.1 kg, U, n = 21) or normal birth weight (1.4–1.6 kg, N, n = 21) were subjected to a feed restriction period (60% of controls with ad libitum consumption) from d 78 until d 98 of age. Subsequently, both restricted (UR, NR) and control groups (UK, NK) were subjected to ad libitum feeding until d 131 (“Refeeding” period). Thus, four groups (NK, NR, UK, UR) were finally compared with each other at each time point (d 98, d 104 and d 131). With the exception of birth weight (d 0), no significant differences in body weight were found between groups.

As a basic requirement of the study, U and N grouped animals (21 animals each) used for liver tissue studies differed in birth weight (d 0) with 1.07±0.02 and 1.56±0.02 kg, respectively (p≤0.001) (Table 2, Figure 1). At later time points after birth (d 75, d 98, d 104 and d 131), no differences in body weight were found between groups (Table 2, Figure 2, n = 3). Accordingly, no correlation was observed between slaughter weight and birth weight of animals (data not shown; p>0.1). However, after five weeks of refeeding (d 131) UK animals tended to have higher body weights as compared to UR, whereas we could not observe differences between NR and NK pigs (p<0.1, Table 2, Figure 2).

**Table 2:** Effects of 3-week feed restriction (age d 98), 5-day (age d 104) and 5-weeks refeeding (age d 131) on body weight development in U and N pigs used for liver tissue studies.

		Body weight [kg; means ± SEM]§			
	d 0#	d 75	d 98	d 104	d 131
**UK**	1.07±0.02***	27.8±2.9	31.3±2.0	38.0±2.4	71.3±0.7
**UR**			27.0±3.5	37.5±3.2	63.4±2.6
**NK**	1.56±0.02	31.3±1.5	29.3±1.6	39.0±6.2	64.9±4.9
**NR**			33.5±2.0	35.4±1.3	68.0±3.4

*** P≤0.001 between groups per time point,

# n = 21 per group;

§ n = 3 per group.

### Effects of low birth weight on hepatic gene expression profile and lipid droplet formation

**Figure 3:**
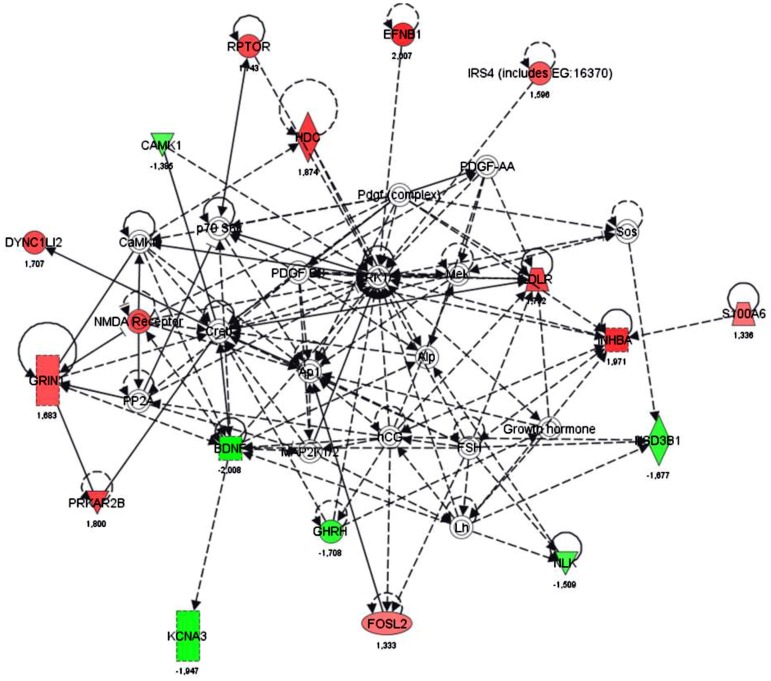
IPA network of differentially regulated genes in the liver of U and N pigs at d 75. Based on IPA (Ingenuity pathway analysis) network analysis, the identified 194 genes sensitive to low (vs. normal) birth weight play a major role in processes of cell death, cellular growth and proliferation, and lipid metabolism. green, down-regulated genes; red, up-regulated genes.

**Figure 4:**
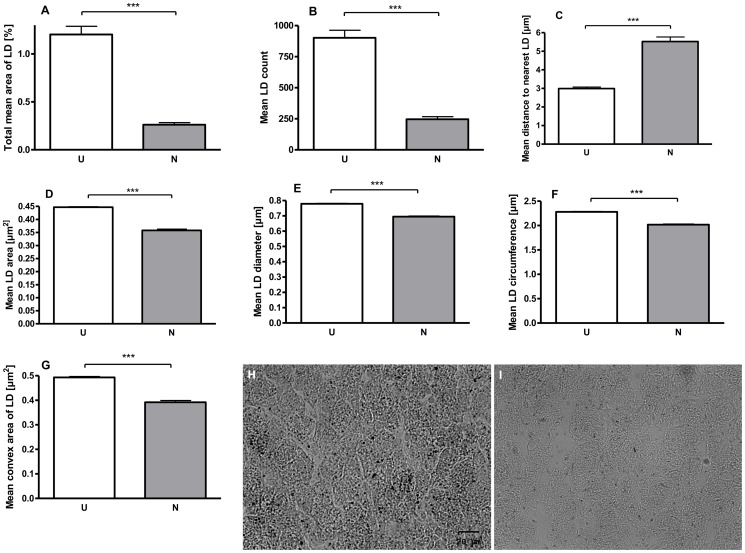
Effects of U and N on liver lipid droplet (LD) count and formation in pigs at d 75. As analyzed by oil red o staining, the total mean area of LDs was increased about 4.6-fold in U vs. N animals (A) and was in agreement with a 3.7-fold higher mean LD count (B). This was also reflected by a 1.85-fold reduction in distance (µm) to nearest LD (C). Moreover, the mean size (µm2) of each LD was increased about 24.9% in U compared to N animals (D). This observation was supported by mean increases of LD diameter (µm) (E), LD circumference (µm) (F) and LD convex area (µm2) (G) of 12.1%, 13.0% and 25.8%, respectively. Representative microscopic images of oil-red o stained liver sections of U (H) and N (I) animals are additionally shown.

Microarray-based whole genome expression profiles were analyzed in liver samples of low (U) and normal (N) birth weight pigs. Based on our selection criteria (fold change levels ≥1.3, p≤0.05), 194 genes (95 up- and 99 down-regulated) were identified to be differentially expressed in the liver of U vs. N pigs at the age of 75 d (prior to feed restriction). IPA (Figure 3) shows a key role for the regulated genes in processes of cell death, cellular growth and proliferation, and lipid metabolism. Since 20% of the strongest regulated genes (EIF2A, HPGDS, PLCG2, CYP7A1) were related to lipid metabolism (Table 3), effects on lipid droplet (LD) count and formation were analyzed in liver tissues of U and N pigs. As shown in Figure 4A, the total mean area of LDs was 4.6-fold higher in U compared to N animals at the age of 75 d. Furthermore, a 3.7-fold higher LD count has been observed, which was also reflected by a reduced distance (1.85-fold) to nearest LD (µm) in U vs. N pigs (Figure 4B–C). In addition, the LD area (µm^2^) was 24.9% higher in U compared to N animals (Figure 4D). This observation was confirmed by larger LD diameter (µm), LD circumference (µm) and LD convex area (µm^2^) of 12.1%, 13.0% and 25.8%, respectively (Figure 4E–G). Assuming a typical round shape of lipid droplets, the volume of LD was calculated with 0.248 and 0.176 µm^3^ for U and N animals, respectively. Taking into account the LD count for equal sized areas, the volume per total LD area was 5.2-fold higher with 223.6 and 43.4 µm^3^ for U and N animals, respectively.

**Table 3:** “Top 10” regulated genes in the liver of 75 d old pigs with low (U) and normal (N) birth weight.

Ensembl ID	Gene symbol	Gene name	Gene Ontology function or process associations	Fold Change
***up-regulated***				
ENSP00000351206	TXLNB	taxilin beta	-	+3.24
ENSP00000389272	RP11-88G17.6	Novel protein similar to hemicentin	-	+3.06
ENSP00000356849	POGK	pogo transposable element with KRAB domain	DNA binding, multicellular organismal development	+2.68
ENSP00000361580	ZNF691	zinc finger protein 691	DNA and metal ion binding	+2.41
ENSP00000273435	EIF2A	eukaryotic translation initiation factor 2A, 65 kDa	tRNA binding, SREBP-mediated signaling pathway	+2.31
ENSP00000394670	PPIA	peptidylprolyl isomerase A (cyclophilin A)	Unfolded protein binding, protein peptidyl-prolyl isomerization, blood coagulation	+2.08
ENSP00000315662	IQSEC3	IQ motif and Sec7 domain 3	ARF guanyl-nucleotide exchange factor activity, regulation of ARF protein signal transduction	+2.08
ENSP00000295256	HPGDS	hematopoietic prostaglandin D synthase	calcium ion binding, fatty acid biosynthetic process, prostaglandin metabolic process	+2.04
ENSP00000388194	RSPO1	R-spondin homolog (Xenopus laevis)	Protein binding, positive regulation of canonical Wnt receptor signaling pathway	+2.02
ENSP00000204961	EFNB1	ephrin-B1	Ephrin receptor and protein binding, cell differentiation, multicellular organismal development, positive regulation of T cell proliferation	+2.01
***down-regulated***				
ENSP00000352336	PLCG2	phospholipase C, gamma 2 (phosphatidylinositol-specific)	Hydrolase activity, phosphatidylinositol phospholipase C activity, Wnt recpeptor signaling pathway, B and T cell receptor signaling pathway, lipid and phospholipid catabolic process	−3.03
ENSP00000301645	CYP7A1	cytochrome P450, family 7, subfamily A, polypeptide 1	Cholesterol 7-alpha-monooxygenase activity, bile acid biosynthetic process, cellular lipid metabolic process, cellular response to cholesterol, cholesterol catabolic process, cholesterol homeostasis	−2.84
ENSP00000401437	LDB3	LIM domain binding 3	Protein and metal ion binding,	−2.77
ENSP00000342518	SLC35F4	solute carrier family 35, member F4	-	−2.69
ENSP00000326324	LRRC37A	leucine rich repeat containing 37A	-	−2.68
ENSP00000410447	IKZF2	IKAROS family zinc finger 2 (Helios)	DNA and metal ion binding, regulation of DNA-dependent transcription	−2.44
ENSP00000348019	SLC17A5	solute carrier family 17 (anion/sugar transporter), member 5	Sialic acid transmembrane transporter activity, sugar∶hydrogen symporter activity, anion transport	−2.43
ENSP00000402584	MDGA1	MAM domain containing glycosylphosphatidylinositol anchor 1	multicellular organismal development, cell differentiation, brain development	−2.41
ENSP00000316740	TTC29	tetratricopeptide repeat domain 29	Binding	−2.35
ENSP00000357844	SLC16A10	solute carrier family 16, member 10	(aromatic) amino acid and transmembrane transport, ion transport	−2.33

In summary, low birth weight induces distinct changes in the metabolic gene expression profile, which was reflected by higher hepatic lipid droplet count and size (Figure 4H–I).

### Influence of feed restriction (R) on gene expression, lipid droplet count and size in the liver of U and N pigs

**Figure 5:**
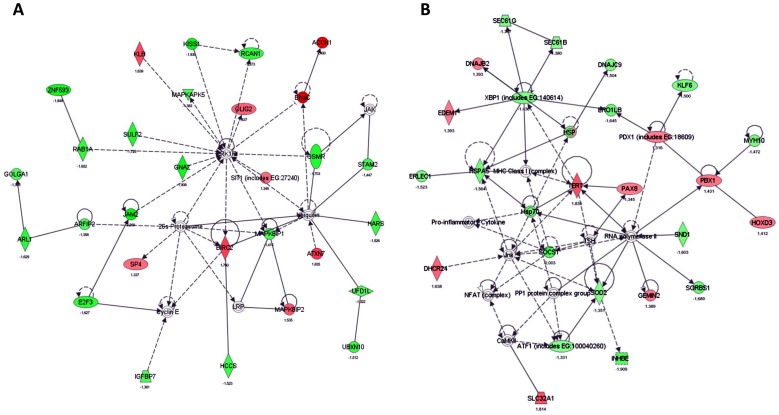
In silico analysis of regulated genes sensitive to feed restriction in N (A) and U pigs (B) via IPA analysis. In liver tissues of pigs with normal birth weight (N), IPA network tool identified regulated genes sensitive to feed restriction with a primary role in cell death, cellular growth and proliferation, and tissue development processes (A). In low birth weight pigs (U), a major role in processes of cell death, protein degradation and protein synthesis was identified (B).

**Figure 6:**
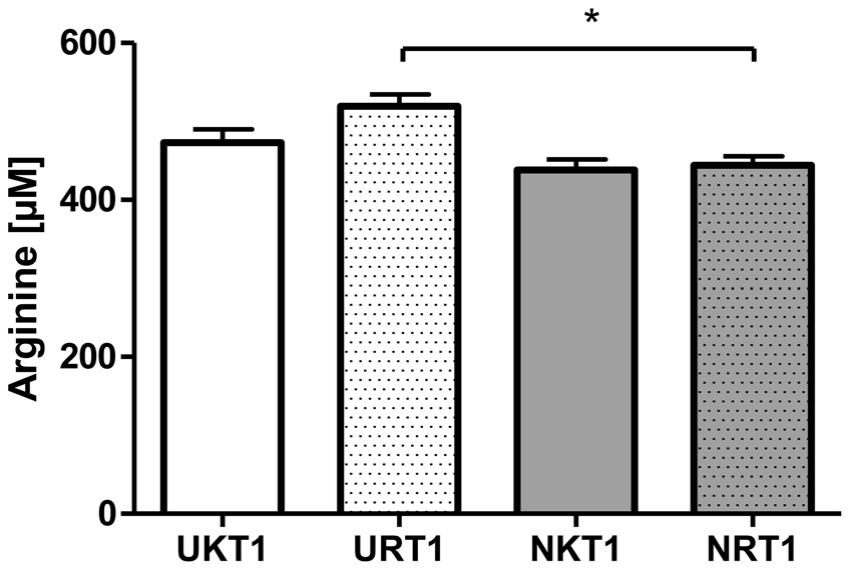
Effects of feed restriction on liver arginine levels in U and N pigs. With regard to birth weight, arginine concentration was significantly increased by about 17% in liver samples of low (UR) vs. normal birth weight (NR) pigs after feed restriction. No significant concentration differences were found for other amino acids.

The influence of the 3-week feed restriction period was analyzed in regard to gene expression and lipid droplet formation in low (U) and normal (N) animals at the age of d 98 (Table 4). In N animals, IPA indicated an involvement of the regulated genes in processes of cell death, cellular growth and proliferation, and tissue development (Figure 5A). Moreover, the affected genes seem to play a role, at least in part, in proteasome-mediated protein degradation. In detail, 31 transcripts of the 451 differentially regulated genes (NR vs. NK, 311 up- and 140 down-regulated) were associated with functional roles in protein and/or amino acid metabolism (Table 5). Of note, about 22.6% of these transcripts (TARSL2, VARS, HARS, EPRS, LARS, FARSB) belong to the group of aminoacyl-tRNA synthetases. Furthermore, numerous transcripts indicate functional roles in arginine (ASS1, SFRS3, SFRS6, PRMT7), tyrosine (TNK2, PTPRS, YWHAB, TTRAP) and serine (PSPH, PRSS55, SFRS3, SFRS6) metabolism. Effects on protein and/or amino acid metabolism were also apparent in U animals after feed restriction (Table 6). Thus, the generated IPA network shows for the 340 differentially regulated genes (UR vs. UK, 121 up- and 219 down-regulated) a functional role in cell death, protein degradation and protein synthesis (Figure 5B). An involvement of these genes (XBP1, SEC61G, SEC61B) in processes of ER stress-associated protein degradation and unfolded protein response (UPR) is also shown in Table 6. Additionally, the regulated genes seem to play a role in inflammatory processes (Figure 5B, Table 7). To analyze the transcriptional effects of feed restriction also on the metabolic level, amino acid concentrations were additionally determined. We could show that the free arginine concentration in the liver of UR animals was 17% higher than in NR (Figure 6). Indeed, no differences were found for other amino acids. Effects of feed restriction were also observed in regard to genes involved in lipid metabolism in N (e.g. ABCA9, FADS1, CROT, STARD13, SC5DL, ACAD10) and U (e.g. CYP26A1, ATP8A2, DHCR24, ACOX3, SLC27A3, HNF4A) animals. Of note, these effects were accompanied by changes in lipid droplet (LD) count and formation. As summarized in Table 4, the total mean area of LD was reduced by 58.3% and 72.7% after feed restriction in U and N animals, respectively. Moreover, parameters of LD formation including mean droplet area (µm^2^), mean LD diameter (µm) and mean LD circumference (µm) were also reduced by 7.9%, 4.7% and 5.6%, respectively, in U animals after feed restriction (UR) (p≤0.05). Significant effects, although of lesser magnitude, were also found in N animals after 3-week feed restriction period (Table 4). However, when related to NK pigs, UK pigs showed higher levels of mean LD area, mean LD diameter and mean LD circumference of about 21.7%, 7.1% and 9.0%, respectively, but LD total mean area was not affected (Table 4).

**Table 4:** Effects of 3-weeks feed restriction (R, T2) on LD size in the liver of U and N pigs.

	Total mean area of LD [%]			Mean droplet size [µm^2^]			LD diameter [µm]			LD circumference [µm]		
vs.	UKT2	URT2	NRT2	UKT2	URT2	NRT2	UKT2	URT2	NRT2	UKT2	URT2	NRT2
**UKT2**	-	−58.3	ns	-	−7.9	ns	-	−4.7	ns	-	−5.6	ns
**URT2**	ns	-	ns	ns	-	ns	ns	-	ns	ns	-	ns
**NKT2**	ns	ns	−72.7	+21.7	ns	ns	+7.1	ns	−3.1	+9.0	ns	−3.3
**NRT2**	ns	ns	-	ns	+18.4	-	ns	+5.2	-	ns	+6.4	-

Reading example URT2 vs. UKT2: The total mean area of LD% in URT2 pigs was reduced by 58.3% as compared to UKT2. Values are expressed as % deviation from the age-matched control groups (i.e. UR vs. UK, UR vs. NR, and NR vs. NK) (p≤0.05) (n = 3 per group).

Non-significant differences among groups are indicated by ‘ns’.

**Table 5:** Identification of differentially regulated genes involved in amino acid metabolism in the liver of N pigs after 3 weeks of feed restriction (age d 98).

Ensembl ID	Gene symbol	Gene name	Gene Ontology function or process associations	Fold Change
***up-regulated***				
ENSP00000388036	ASPHD1	aspartate beta-hydroxylase domain containing 1	peptidyl-amino acid modification	+2.82
ENSP00000217420	SLC32A1	solute carrier family 32 (GABA vesicular transporter), member 1	gamma-aminobutyric acid∶hydrogen symporter activity, glycine transmembrane transporter activity	+2.40
ENSP00000378854	PSPH	phosphoserine phosphatase	L-serine biosynthetic process, phosphoserine phosphatase activity	+1.71
ENSP00000333003	PRSS55	protease, serine, 55	serine-type endopeptidase activity	+1.67
ENSP00000317836	PRR15	proline rich 15	multicellular organismal development	+1.54
ENSP00000368135	LRRN4	leucine rich repeat neuronal 4	-	+1.52
ENSP00000280527	CRIM1	cysteine rich transmembrane BMP regulator 1 (chordin-like)	regulation of cell growth, insulin-like growth factor binding, serine-type endopeptidase inhibitor activity	+1.49
ENSP00000320324	NPEPPS	aminopeptidase puromycin sensitive	cytosol alanyl aminopeptidase activity,	+1.49
ENSP00000361470	ASS1	argininosuccinate synthase 1	Arginine biosynthesis, Amino-acid biosynthesis, urea cycle	+1.47
ENSP00000413373	TNK2	tyrosine kinase, non-receptor, 2	protein autophosphorylation	+1.47
ENSP00000329291	TARSL2	threonyl-tRNA synthetase-like 2	threonyl-tRNA aminoacylation, threonine-tRNA ligase activity	+1.36
ENSP00000348043	LRRC20	leucine rich repeat containing 20	-	+1.36
***down-regulated***				
ENSP00000317382	SLC36A4	solute carrier family 36 (proton/amino acid symporter), member 4	L-alanine transport, proline transport, tryptophan transport	−2.18
ENSP00000319255	CHORDC1	cysteine and histidine-rich domain (CHORD)-containing 1	Stress response	−2.02
ENSP00000250237	QTRT1	queuine tRNA-ribosyltransferase 1	Queuosine biosynthesis, tRNA processing	−1.93
ENSP00000349932	PTPRS	protein tyrosine phosphatase, receptor type, S	peptidyl-tyrosine dephosphorylation	−1.76
ENSP00000344762	SFRS3	serine/arginine-rich splicing factor 3	insulin receptor signaling pathway, mRNA processing, RNA splicing	−1.62
ENSP00000244020	SFRS6	serine/arginine-rich splicing factor 6	RNA binding, nucleotide binding, negative regulation of nuclear mRNA splicing, via spliceosome	−1.59
ENSP00000401121	VARS	valyl-tRNA synthetase	valine-tRNA ligase activity, Aminoacyl-tRNA synthetase	−1.55
ENSP00000413162	VARS	valyl-tRNA synthetase	valine-tRNA ligase activity, Aminoacyl-tRNA synthetase	−1.54
ENSP00000425634	HARS	histidyl-tRNA synthetase	histidine-tRNA ligase activity, histidyl-tRNA aminoacylation	−1.53
ENSP00000355890	EPRS	glutamyl-prolyl-tRNA synthetase	proline-tRNA ligase activity, glutamate-tRNA ligase activity	−1.50
ENSP00000274562	LARS	leucyl-tRNA synthetase	leucine-tRNA ligase activity, leucyl-tRNA aminoacylation	−1.49
ENSP00000281828	FARSB	phenylalanyl-tRNA synthetase, beta subunit	phenylalanine-tRNA ligase activity, phenylalanyl-tRNA aminoacylation	−1.47
ENSP00000361930	YWHAB	tyrosine 3-monooxygenase/tryptophan 5-monooxygenase activation protein, beta polypeptide	monooxygenase activity, protein targeting	−1.42
ENSP00000367440	TTRAP	tyrosyl-DNA phosphodiesterase 2	5′-tyrosyl-DNA phosphodiesterase activity, transcription corepressor activity, protein binding	−1.42
ENSP00000303754	PPID	peptidylprolyl isomerase D	peptidyl-prolyl cis-trans isomerase activity, protein folding	−1.39
ENSP00000409324	PRMT7	protein arginine methyltransferase 7	cell differentiation, DNA methylation involved in gamete generation, regulation of gene expression by genetic imprinting	−1.38
ENSP00000278723	GRIK4	glutamate receptor, ionotropic, kainate 4	kainate selective glutamate receptor activity, synaptic transmission, extracellular-glutamate-gated ion channel activity	−1.34
ENSP00000359557	LDOC1	leucine zipper, down-regulated in cancer 1	-	−1.33
ENSP00000264094	LOXL3	lysyl oxidase-like 3	oxidoreductase activity, acting on the CH-NH2 group of donors, oxygen as acceptor, scavenger receptor activity	−1.31

**Table 6:** Identification of differentially regulated genes involved in amino acid metabolism in the liver of U pigs after 3 weeks of feed restriction (age d 98).

Ensembl ID	Gene symbol	Gene name	Gene Ontology function or process associations	Fold Change
***up-regulated***				
ENSP00000217420	SLC32A1	Solute carrier family 32 (GABA vesicular transporter), member 1	glycine transmembrane transporter activity	+1.81
ENSP00000409976	ASPA	Aspartoacyclase	hydrolase activity, acting on carbon-nitrogen (but not peptide) bonds, in linear amides	+1.77
ENSP00000013222	INMT	Indolethylamine N-methyltransferase	methyltransferase activity	+1.48
ENSP00000310447	GLS2	Glutaminase 2	glutamine metabolic process, glutaminase activity	+1.41
ENSP00000325589	PTPRCAP	protein tyrosine phosphatase, receptor type, C-associated protein	receptor activity	+1.33
***down-regulated***				
ENSP00000355632	GALNT2	UDP-N-acetyl-alpha-D-galactosamine∶polypeptide N-acetylgalactosaminyltransferase 2	protein O-linked glycosylation via serine or threonine	−2.03
ENSP00000356207	PPFIA4	protein tyrosine phosphatase, receptor type, f polypeptide (PTPRF), interacting protein (liprin), alpha 4	Protein binding	−1.92
ENSP00000296486	AGXT2L1	alanine-glyoxylate aminotransferase 2-like 1	cellular amino acid metabolic process, alanine-glyoxylate transaminase activity	−1.89
ENSP00000278360	PAMR1	peptidase domain containing associated with muscle regeneration 1	serine-type endopeptidase activity	−1.60
ENSP00000332223	CRELD2	cysteine-rich with EGF-like domains 2	Protein binding, calcium ion binding	−1.60
ENSP00000324173	HSPA5	heat shock 70 kDa protein 5 (glucose-regulated protein, 78 kDa)	ER overload response, ER-associated protein catabolic process, regulation of protein folding in ER	−1.56
ENSP00000379250	AURKA	aurora kinase A	protein serine/threonine/tyrosine kinase activity	−1.51
ENSP00000391739	PISD	phosphatidylserine decarboxylase	phosphatidylserine decarboxylase activity	−1.49
ENSP00000331368	SERPINB8	serpin peptidase inhibitor, clade B (ovalbumin), member 8	serine-type endopeptidase inhibitor activity, protein binding	−1.48
ENSP00000384295	XBP1	X-box binding protein 1	activation of signaling protein activity involved in unfolded protein response, positive regulation of endoplasmic reticulum unfolded protein response, immune response	−1.43
ENSP00000366156	SRM	spermidine synthase	polyamine metabolic process	−1.40
ENSP00000341538	SEC61G	Sec61 gamma subunit	P-P-bond-hydrolysis-driven protein transmembrane transporter activity, SRP-dependent cotranslational protein targeting to membrane	−1.39
ENSP00000387180	PTRH2	peptidyl-tRNA hydrolase 2	aminoacyl-tRNA hydrolase activity	−1.38
ENSP00000223641	SEC61B	Sec61 beta subunit	ER-associated protein catabolic process, retrograde protein transport, ER to cytosol	−1.38
ENSP00000250237	QTRT1	queuine tRNA-ribosyltransferase 1	queuine tRNA-ribosyltransferase activity	−1.34

**Table 7:** Identification of differentially regulated genes involved in inflammatory processes in the liver of U pigs after 3 weeks of feed restriction (age d 98).

Ensembl ID	Gene symbol	Gene name	Gene Ontology function or process associations	Fold Change
***up-regulated***				
ENSP00000225842	CCL1	chemokine (C-C motif) ligand 1	Chemokine activity, immune response	+2.37
ENSP00000269485	TNFRSF11A	tumor necrosis factor receptor superfamily, member 11a, NFKB activator	Adaptive immune response, response to cytokine stimulus, positive regulation of NF-kappaB transcription factor activity	+2.25
ENSP00000386239	NFKBIL2	nuclear factor of kappa light polypeptide gene enhancer in B-cells inhibitor-like 2	histone binding, protein binding, transcription corepressor activity	+1.62
ENSP00000399105	IL2RB	interleukin 2 receptor, beta	cytokine-mediated signaling pathway, interleukin-2 receptor activity, interleukin-2 binding	+1.40
ENSP00000398379	STAT5B	signal transducer and activator of transcription 5B	T cell homeostasis, cytokine-mediated signaling pathway, isoleucine metabolic process, liver development	+1.40
ENSP00000241052	CAT	catalase	NADP binding, catalase activity, oxidoreductase avtivity, aminoacylase activity	+1.39
ENSP00000420269	ADH7	alcohol dehydrogenase 7	oxidoreductase activity	+1.38
***down-regulated***				
ENSP00000366729	TNFRSF9	tumor necrosis factor receptor superfamily, member 9	induction of apoptosis, receptor activity	−3.82
ENSP00000247668	TRAF2	TNF receptor-associated factor 2	activation of NF-kappaB-inducing kinase activity, positive regulation of T cell activation, innate immune response, tumor necrosis factor-mediated signaling pathway	−2.31
ENSP00000329418	SOCS1	suppressor of cytokine signaling 1	type I interferon-mediated signaling pathway, interferon-gamma-mediated signaling pathway	−2.00
ENSP00000394473	DOCK8	dedicator of cytokinesis 8	blood coagulation	−1.87
ENSP00000371676	IFNAR2	interferon (alpha, beta and omega) receptor 2	type I interferon binding, type I interferon receptor activity	−1.78
ENSP00000355046	MT-ND2	mitochondrially encoded NADH dehydrogenase 2	NADH dehydrogenase (ubiquinone) activity, mitochondrial electron transport, NADH to ubiquinone	−1.74
ENSP00000368698	HIVEP1	human immunodeficiency virus type I enhancer binding protein 1	zinc ion binding	−1.65
ENSP00000416956	PDIA5	protein disulfide isomerase family A, member 5	protein disulfide oxidoreductase activity, electron carrier activity	−1.49
ENSP00000376500	TRAF3	TNF receptor-associated factor 3	innate immune response, toll-like receptor signaling pathway, tumor necrosis factor-mediated signaling pathway	−1.41
ENSP00000376092	CASP8	caspase 8, apoptosis-related cysteine peptidase	response to tumor necrosis factor, innate immune response, positive regulation of I-kappaB kinase/NF-kappaB cascade	−1.37
ENSP00000337127	SOD2	superoxide dismutase 2, mitochondrial	removal of superoxide radicals, age-dependent response to reactive oxygen species, oxygen homeostasis	−1.35
ENSP00000355190	NFE2L1	nuclear factor (erythroid-derived 2)-like 1	inflammatory response, heme biosynthetic process	−1.35
ENSP00000262053	ATF1	activating transcription factor 1	innate immune response, Toll signaling pathway, stress-activated MAPK cascade	−1.33

**Figure 7:**
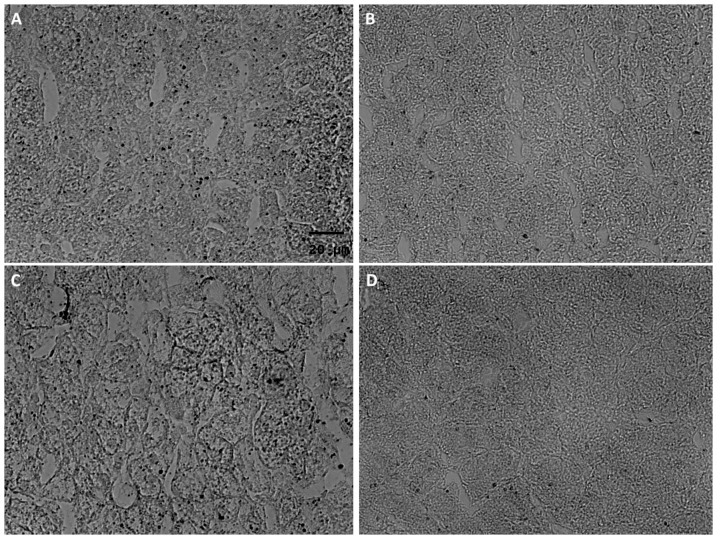
Microscopic images of oil red O stained lipid droplets in the liver of feed-restricted (R) and non-restricted (K) U and N pigs. Feed restriction induced a distinct decrease of the total mean area of LDs in both low (B vs. A) and normal (D vs. C) birth weight pigs. This was also supported by detailed analysis of liver tissue samples.

In summary, feed restriction induced changes in the hepatic gene expression profile that was translated into increased free liver arginine levels in U animals. Moreover, LD numbers and formation were also reduced in U and N animals during feed restriction (Figure 7A–D).

### Long-term effects of feed restriction on the molecular and physiological level in U and N pigs

**Figure 8:**
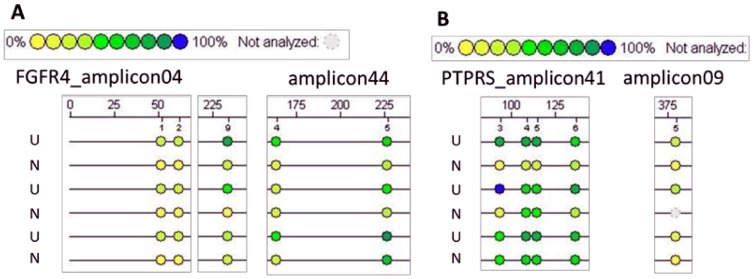
Promoter methylation analysis of FGFR4 and PTPRS gene. As shown in Figure A, low birth weight (U) induced increased methylation status of FGFR4 gene in amplicons 04 (#1and #2 = −2635_−263 and −2625_−2624 and #9 = −2453_−2452) and amplicon 44 (#4 = −1046_−1045 and #5 = −1109_−1108) when related to normal birth weight animals (N). This was supported by significant decreases in gene expression (FC = −1.36, p = 0.018, data not shown). An increased methylation status with regard to birth weight was also indicated in PTPRS gene promoter (B) (amp41 #3 = −477_−476, #4 and #5 = −492_−491 and −498_−497, #6 = −520_−519; amp09 #5 = −1818_−1817), however, with no changes in gene expression (FC = −1.44, p = 0.213, data not shown).

**Figure 9:**
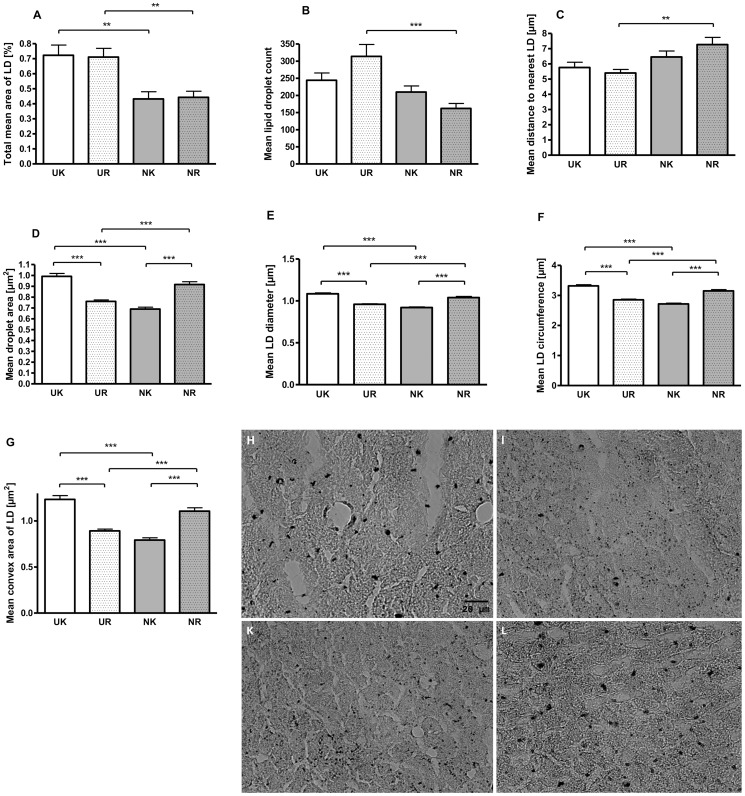
Effect of refeeding period on lipid droplet (LD) count and formation in previously feed restricted (R) and non-restricted (K) N and U pigs. As shown in Figure A, 5-week refeeding period induced the recovery of total mean LD area in previously feed-restricted animals (UR, NR) when compared to their non-restricted age matched controls (UK, NK). Moreover, LD count was increased by 51.6% in UR vs. NR animals (B), which was accompanied by a decreased mean distance to nearest LD (C). In contrast, the mean lipid droplet size (µm^2^) was reduced by 23.3% in UR vs. UK animals (D). This was supported by decreases of mean LD diameter (E), mean LD circumference (F), and mean convex area (G) of 11.5%, 14.0% and 27.8%, respectively. Of note, in N animals, the opposite effect was observed. Here, the mean LD size (µm2) was increased by 24.7% when compared to controls (NK) (D). This finding was supported by increases of LDs in mean diameter (E), mean circumference (F) and mean convex area (G) of 11.5%, 13.7% and 28.4%, respectively. These results are illustrated by representative microscopic observations of oil-red stained liver sections of UK (H), UR (I), NK (K) and NR (L) pigs.

To further analyze immediate effects of feed restriction that might persist after refeeding, previously feed-restricted (UR, NR) and age-matched non-restricted control animals (UK, NK) were fed *ad libitum* for another 5 weeks until the age of d 131. Subsequently, effects on gene expression and metabolic variables related to amino acid and lipid metabolism were analyzed. As shown in Table 8 and 9, 4 (2 up- and 2 down-regulated) and 22 genes (4 up- and 18 down-regulated) show persistent regulation in U and N animals after 5 weeks of refeeding, respectively. These genes are involved in processes of lipid (FABP5, WNT5B, ACSL5, FGFR4) and protein metabolism (PTPRS, HSPA8, FGFR4), glucose homeostasis (SORT1, PPP1R3E, FGFR4) and (post)transcriptional modification (NFKBIL2, CC2D1A, NSUN2, CAND2, PRMT7). In view of the fact that long-term transcriptional changes may be correlated with CpG island variation [31], methylation analyses were conducted in putative promoter regions of persistently regulated genes. Finally, 7 relevant metabolic genes (PTPRS, WNT5B, FABP5, PPP1R3E, PDE9A, SORT1, FGFR4) were included in methylation analysis. As shown in Figure 8 A and B, effects were found for FGFR4 and PTPRS gene with regard to birth weight (U vs. N). Thus, in U pigs, regions with a tendency of increased methylation where identified for FGFR4 and PTPRS. In case of PTPRS CpG-sites with increased methylation ratio in “U”-pigs are located in regions −2186 to −1700 and −1033 to −730 relative to mRNA/gene start. In case of FGFR4, regions −2675 to 2178 and −1392 to −893 display the methylation change. Of note, FGFR4 gene expression was also reduced (FC = −1.36, p = 0.018). No transcriptional effects have been found for PTPRS gene (FC = −1.44, p = 0.213). Further effects of refeeding were analyzed in regard to LD formation and free amino acid concentrations in the liver of U and N animals. As shown in Figure 9A, the 5-weeks refeeding period induced the recovery of total mean LD area (%) in previously feed-restricted U and N animals (UR, NR) when related to their age-matched non-restricted controls (UK, NK). The LD count was greater (+51.6%, p≤0.001) in UR compared to NR animals (Figure 9B), which was accompanied by a decreased distance to nearest LD (Figures 9C). However, the LD size (µm^2^) was reduced by 17.1% in UR vs. NR animals, and by 23.3% in UR vs. UK animals (Figure 9D). This finding was supported by decreases in LD diameter and circumference, and LD convex area of 11.5%, 14.0% and 27.8% in UR vs. UK animals, respectively (Figures 9E–G). Indeed, in N animals, the opposite effect was observed. Thus, in NR pigs, the LD size (µm^2^) was increased by 24.7% when compared to NK (Figure 9D). This finding was supported by increases in LD diameter, LD circumference and convex LD area of 11.5%, 13.7% and 28.4%, respectively (Figures 9E–G).

**Table 8:** Identification of long-term regulated genes in the liver of U pigs responsive to 3-week feed restriction (T2, age d 98) and after 5 weeks of refeeding (T3, age d 131).

Ensembl ID	Gene symbol	Gene name	Gene Ontology function or process associations	Fold Change	
				URT2 vs. UKT2	URT3 vs. UKT3
***up-regulated***					
ENSP00000374854	IGLV3-1	immunoglobulin lambda variable 3-1	-	+1.82	+2.07
ENSP00000386239	NFKBIL2	Inhibitor of kappa B-related protein	histone binding, transcription corepressor activity	+1.62	+1.42
***down-regulated***					
ENSP00000281924	TMEM163	transmembrane protein 163	cation transmembrane transporter activity	−1.89	−2.01
ENSP00000355046	MT-ND2	NADH-ubiquinone oxidoreductase chain 2	NADH dehydrogenase activity, small molecule metabolic process	−1.74	−1.33

**Table 9:** Identification of long-term regulated genes in the liver of N pigs responsive to 3-week feed restriction (T2, age d 98) and after 5 weeks of refeeding (T3, age d 131).

Ensembl ID	Gene symbol	Gene name	Gene Ontology function or process associations	Fold Change	
				NRT2 vs. NKT2	NRT3 vs. NKT3*
***up-regulated***					
ENSP00000263369	MIA	melanoma inhibitory activity	growth factor activity	+1.63	+3.99
ENSP00000313601	CC2D1A	coiled-coil and C2 domain containing 1A	regulation of transcription, DNA-dependent, signal transducer activity	+1.58	+1.79
ENSP00000354481	FAM5B	family with sequence similarity 5, member B	-	+1.42	+1.85
ENSP00000418356	MRAS	muscle RAS oncogene homolog	GTP binding, GTPase activity	+1.48	+1.97
***down-regulated***					
ENSP00000256637	SORT1	sortilin 1	glucose import, response to insulin stimulus, negative regulation of apoptotic process	−1.82	−1.94
ENSP00000230050	RPS12	ribosomal protein S12	-	−1.38	−1.60
ENSP00000264670	NSUN2	NOP2/Sun domain family, member 2	tRNA binding, tRNA (cytosine-5-)-methyltransferase activity	−1.53	−1.37
ENSP00000297258	FABP5	fatty acid binding protein 5	fatty acid binding, protein binding, transporter activity	−1.32	−1.70
ENSP00000308887	WNT5B	wingless-type MMTV integration site family, member 5B	Positive regulation of fat cell differentiation and cell migration, cellular response to retinoic acid	−1.62	−1.92
ENSP00000322061	C7	complement component 7	Complement activation, cytolysis	−1.48	−1.74
ENSP00000346001	RPL3	small nucleolar RNA, C/D box 43	translation	−1.33	−1.35
ENSP00000349932	PTPRS	protein tyrosine phosphatase, receptor type, S	transmembrane receptor protein tyrosine phosphatase activity, protein binding	−1.76	−1.64
ENSP00000354717	PPP1R3E	protein phosphatase 1, regulatory (inhibitor) subunit 3E	glycogen metabolic process	−1.45	−1.66
ENSP00000355688	GUK1	guanylate kinase 1	purine nucleotide metabolic process, nucleobase-containing small molecule interconversion	−1.44	−1.35
ENSP00000362524	ANGPTL2	angiopoietin-like 2	multicellular organismal development, signal transduction	−1.35	−1.46
ENSP00000364240	UBXN10	UBX domain protein 10	-	−1.51	−1.61
ENSP00000376526	HSPA8	heat shock 70 kDa protein 8	protein folding and binding, response to unfolded protein, negative regulation of transcription, DNA-dependent	−1.89	−1.94
ENSP00000376796	ACSL5	acyl-CoA synthetase long-chain family member 5	long-chain fatty acid-CoA ligase activity, ATP binding, fatty acid transport, response to cholesterol and glucose stimulus	−1.36	−1.44
ENSP00000381280	PDE9A	phosphodiesterase 9A	3′,5′-cyclic-GMP phosphodiesterase activity, blood coagulation, metal ion and protein binding	−1.52	−1.52
ENSP00000387641	CAND2	cullin-associated and neddylation-dissociated 2 (putative)	Positive regulation of transcription, DNA-dependent	−1.60	−1.35
ENSP00000409324	PRMT7	protein arginine methyltransferase 7	DNA methylation involved in gamete generation, regulation of gene expression by genetic imprinting, cell differentiation, regulation of protein binding	−1.38	−1.42
ENSP00000366412	FGFR4	fibroblast growth factor receptor 4	glucose homeostasis, peptidyl-tyrosine phosphorylation, regulation of cholesterol homeostasis, positive regulation of proteolysis	−1.41*	−1.52

* p≤0.05 or the highest absolute signal in one group is equal or lower than the lowest value in the other group and vice versa.

With regard to the observed effects of feed restriction on hepatic arginine levels (Figure 6), measurements were also done after a 5-week refeeding period. However, there were not observed any differences later on between UR and NR (data not shown). Thus, in terms of the range of free arginine concentrations it was similar to those observed before feed restriction (R = K).

## Discussion

Studies in animals [32]–[34] and humans [35] show a relationship between IUGR and the risk for metabolic-related diseases in later life. Individuals born with SGA show higher adult abdominal fat mass, lower insulin sensitivity and disturbances of other physiological parameters related to e.g. hepatic lipid metabolism [8], [36]–[38]. Moreover, there is also evidence in the literature that early postnatal dietary restriction may improve metabolic parameters in IUGR offspring [39], [40]. Based on these observations, we used the swine model to determine responses to low birth weight (vs. normal birth weight) after a postnatal feed restriction period and subsequent refeeding on the liver of female juvenile pigs. The swine model was used, because the pig resembles the human physiology in more ways than any other non-primate mammalian species. This is primarily due to physiological and anatomical similarity of the digestive tract [22]. As a basic requirement of our study, birth weights differed significantly between low and normal birth weight animals (1.07±0.02 vs. 1.56±0.02 kg) used for liver tissue analysis. Liver was selected as the target organ due to its primary importance in carbohydrate, protein and lipid metabolism. IPA for the differentially regulated genes showed an involvement in processes of cell death, cellular growth and proliferation, and lipid metabolism. Hence, the two strongest down-regulated genes (PLCG2, CYP7A1) suggest an impact of low birth weight on the regulation of lipid and/or cholesterol metabolism processes [41]–[44]. So far, only few articles have been published for humans and rodents investigating the relationship between low birth weight (IUGR) and hepatic lipid metabolism [9], [45]–[47]. The higher liver fat contents observed in e.g. guinea pigs [45] and rat fetuses [9] with IUGR were attributed to inflammatory responses [46], [48], [49]. In fact, there is strong evidence in the literature supporting a positive relationship between inflammatory stress conditions and lipid and/or cholesterol accumulation in the liver [50]. The higher inflammatory status observed in IUGR animals was accompanied by increased ER stress, finally leading to UPR [51], [52]. In this process, the hydrophobic matrix of LDs has been shown to become a sequestering surface for misfolded proteins [53]. Zhang et al. described the excessive deposition of LDs in cell types such as hepatocytes and macrophages as a hallmark in ER-stress associated metabolic diseases including fatty liver disease [52]. Additionally, Lee et al. showed that ER stress promotes hepatic lipogenesis and LD formation in vitro [51]. Lipid droplets are linked to many cellular functions, including lipid storage for energy generation and membrane synthesis, and protein degradation [54]. Additionally, LD biogenesis is considered a physiological defense mechanism of the liver. Thus, through esterification of free fatty acids and its conversion into triglycerides and LD storage, fatty acid-induced toxicity of cells is reduced [55]. However, to prevent uncontrolled LD expansion, lipolysis becomes activated under physiological conditions. Defects in the regulation of lipid accumulation induce liver steatosis [56]. Autophagy has been identified as the mechanism to regulate the control of hepatic LD growth under pathological conditions. This process is associated to the maintenance of blood glucose and amino acid levels [57]. Hence, the increased LD count and size observed in low birth weight animals (vs. N) in our study could be due, at least in part, to autophagy-related processes.

Energetic restriction is hypothesized to improve metabolic outcomes in IUGR offspring [40]. This assumption was tested in liver tissues of U and N animals after three weeks of feed restriction (d 98). Microarray results indicated 20 transcripts with different expression levels (FC≥1.3, p≤0.05) that have functions in inflammation (Table 6) and UPR response (XBP-1, SEC61B, SEC61G, GRP78 ( = HSPA5)). The X-box binding protein 1 (XBP-1) is required for the function of normal fatty acid synthesis in the liver, and is thus an important regulator of hepatic lipogenesis [58], [59]. Moreover, a study in the liver of adult mice showed that 50% loss of glucose-regulated protein 78 (GRP78) caused an ER stress response, which was accompanied by the onset of apoptosis [60]. Furthermore, these mice exhibited increased fat accumulation in the liver. In agreement with our text mining analysis, the regulated genes in pig liver were attributed to processes of cell death, protein degradation and protein synthesis. Of interest is that these effects were not found in NR pigs. In N animals, the effects were mainly seen in a reduction of mRNA levels of aminoacyl tRNA synthetases and their processing (TARSL2, QTRT1, VARS, HARS, EPRS, LARS, FARSB). Since the presence of aminoacyl tRNA synthetases is a precondition for translation, this finding likely reflects the reduced protein synthesis during feed restriction [61]–[64]. With regard to the observed transcriptional effects of feed restriction on processes related to protein degradation and synthesis, amino acids were additionally determined in liver tissues of U and N animals. We could show that a 3-week feed restriction period induced an increase of free arginine levels in the liver of U pigs as compared to feed-restricted N pigs. Arginine, the nitrogenous precursor of nitric oxide [65] has been shown to regulate multiple metabolic pathways involved in the metabolism of fatty acids, glucose, amino acids, and proteins through cell signaling and gene expression [66]. Of note, arginine is converted to nitric oxide (NO) by nitric oxide synthase (NOS) in almost all mammalian cells [65], [67]. Secondly, NO increases the phosphorylation of hormone-sensitive lipase and perilipins, finally leading to the translocation of the lipase to the neutral LDs, and, hence, the stimulation of lipolysis [68]–[71]. With regard to the results from gene expression and amino acid analysis, diet-induced effects on hepatic LD content were also hypothesized. A 3-week feed restriction period induced a significant decrease in the total mean area of LDs of 58.32 and 72.67% in U and N animals, respectively, when related to their age-matched controls. In general, feed restriction effects were more pronounced on LD count than on LD size in both U and N animals. The observed strong decrease in LD count was also found in liver tissue of caloric-restricted rats when related to *ad libitum* fed animals [72]. Moreover, a recent study in rats showed that early postnatal caloric restriction protected adult male IUGR offspring from obesity [73]. However, there is a lack of studies determining effects of feed restriction and subsequent refeeding in view of birth weight [74]–[76]. The first evidence of how deficient nutrient supply in utero affects birth weight, and the subsequent risk for health disorders in the offspring came from large epidemiological studies such as The Dutch Hunger Winter Families study in 1944–1945 [77] [78], [79]. It was thus assumed that environmental conditions in early life can change epigenetic settings which remain throughout life [80], [81].

The second aim of our pig study was to determine long-term effects of feed restriction on molecular features of hepatic lipid metabolism in relation to birth weight. First of all, significantly regulated genes sensitive to feed restriction, and moreover, with persisting expression levels after five weeks of refeeding, were selected. Overall, the 26 identified genes have functions in DNA-dependent transcription processes, cell differentiation and metabolic homeostasis. Of note, the up-regulated IGLV3-1 gene (in U animals) encodes an antibody of the innate immune system that has been shown to induce tumor-specific cell death via intracellular lipid accumulation, a process that is named lipoptosis [82]. Long-term regulated gene expression is thought to be mediated by epigenetic mechanisms [83]. Epigenetic changes are changes in gene function that occur without changes in gene sequence [84]. Epigenetic regulations can occur at the level of DNA methylation, histone modification or microRNA regulation. DNA methylation of CpG dinucleotides is accompanied by the inability of transcription factors to bind to specific DNA regulatory sequences, and is thus closely linked to silencing of gene transcription. With regard to our study, persistently regulated genes were further selected for methylation analysis. Hence, only genes with detectable CpG island regions in the promoter region, and with publicly available sequence annotations were further considered. With regard to the selected genes (PTPRS, WNT5B, FABP5, PPP1R3E, PDE9A, SORT1, FGFR4), detectable effects were found for FGFR4 and PTPRS genes in relation to birth weight (U vs. N). Thus, U animals showed increased methylation status in each two regions of fibroblast growth factor receptor 4 (FGFR4) and protein tyrosine phosphatase, receptor type, S (PTPRS) gene. In concordance to common opinion, the increased methylation ratio in the FGFR4 gene in U vs. N animals corresponds with a decreased expression rate. A study in FGFR4-deficient mice on a normal diet exhibited features of the metabolic syndrome, including increased mass of white adipose tissue, hyperlipidemia, hypercholesterolemia and insulin resistance [85]. Moreover, restoration of FGFR4 in hepatocytes of FGFR4-deficient mice restored fatty liver with a simultaneous decrease of plasma lipids. Thus, FGFR4 seems to play a role in hepatic lipid metabolism and might explain, at least in part, the increased LD count and size observed in low birth weight animals (U vs. N) in our study. To get an idea about the persistence of metabolic effects related to lipid metabolism in low birth weight animals, previously feed-restricted pigs were also analyzed after five weeks of refeeding. Of specific interest is that the mean LD size increased about 24.7% in NR animals when related to NK animals. Indeed, in U animals the opposite effect was observed after 5 weeks of refeeding. Thus, LD size was 23.3% lower in UR vs. UK animals. This finding was supported by further reductions of parameters related to mean LD size such as diameter, circumference and convex area. In summary, it seems that the 3-week feed-restriction period sensitized U animals for an increased catabolic rate of LDs in the liver. This is due to the fact that the smaller LD droplets observed in the liver of previously feed-restricted U animals are possibly more accessible for surface lipases and thus lipid oxidation processes [86]–[88]. A study in rats showed isomer-specific effects of conjugated linoleic acid (CLA) on LD size [89]. Finally, smaller hepatic LD were associated with overall lower total lipid content within these droplets and improved liver function when compared to larger hepatic droplets. Moreover, the smaller size of LDs was also accompanied by lower hepatic levels of perilipin 2 (PLIN2). Perilipins, the best known LD surface proteins, can either recruit lipases or prevent the access of lipases to LDs [90]. In mammals, five perilipins are known [91]. In our study, perilipin 4 (PLIN4) was reduced about 2.1-fold (p = 0.05) in UR compared to NR animals after 5-weeks of refeeding. In this context, studies in perilipin-knockout mice show an almost complete loss of body fat [92], [93], which was shown to be due to a high basal lipolysis rate. Furthermore, these mice were resistant to diet-induced as well as genetic obesity. Overall, based on our data, we suggest that the 3-week feed-restriction period sensitized UR animals for a higher lipolytic activity in later life. This assumption was supported by the increased expression levels of fatty acid desaturase 2 (FADS2, FC = 2.03, p = 0.03) in UR compared to NR animals after five weeks of refeeding. Thus, when related to NK animals, FADS2 expression was markedly reduced (FC = −1.66, p = 0.067) in NR animals. FADS2 belongs to the group of fatty acid desaturase (FADS) enzymes of the omega 6 family that regulate the unsaturation of fatty acids through the introduction of double bonds [94]. In turn, dietary polyunsaturated fatty acids (PUFA) of the omega 3 and omega 6 family suppress the expression of lipogenic genes while concomitantly inducing the expression of genes related to fatty acid oxidation [95]–[97]. Of further interest are the data from FADS2 knockout mice, which show an increased macrophage cholesterol biosynthesis and decreased cellular paraoxonase 2 (PON2) expression [98]. In our study, PON1 and PON3 have been found to be the strongest down-regulated genes (FC = −11.13 and −5.73, respectively, p≤0.05) in the liver of NR animals when related to their age-matched controls after five weeks of refeeding. Serum PON1 is an HDL-associated lipolactonase, which is synthesized and secreted by the liver [99]. PON1 has antioxidant properties [100], which are associated with the enzyme's capability to prevent oxidation processes in HDL [101] and LDL [102], to decrease the oxidative status in macrophages [103] and atherosclerotic lesions [104], and to stimulate cholesterol efflux from macrophages [105]. Thus, based on PON1 and PON3 expression levels, oxidative stress conditions seem to be increased in previously feed-restricted N animals after five weeks of refeeding. This assumption is supported by data of a mouse model of moderate caloric restriction, in which a 10–15% weight loss that is comparable to human dieting induced increases of the level of the stress hormone plasma corticosterone [106]. Of note, in this study the observed effects on gene expression and promoter methylation of corticotropin-releasing factor were not normalized after refeeding. Moreover, studies in adult rats show that catch-up growth after feed restriction resulted in increased intramuscular and intrahepatic lipid content, visceral fat deposition, and dyslipidaemia [75], [76]. Thus, a link between inflammatory response and hepatic lipid accumulation is suggested. Another study showed that treatment of microglia with pro-inflammatory lipopolysaccharide induced not only accumulation of LDs but also increased their size [107]. This stress-associated response may partly explain the observed increased LD size in previously feed-restricted N animals after refeeding. On the basis of our observation period we conclude that early-life feed restriction may program juvenile female pigs with low birth weight for an increased rate of hepatic lipolysis in later life. Finally, the observed differences in the metabolic responses between low and normal birth weight animals might be due to metabolic imprinting during critical periods of life [17], [108], inter alia mediated by epigenetic mechanisms such as DNA methylation. However, other epigenetic mechanisms such as histone modification and microRNA (miR) regulation may also explain the observed long-term effects [109]. These investigations are planned to be addressed in future research.
